# Exercise induced laryngeal obstruction: a review of diagnosis and management

**DOI:** 10.1007/s00405-016-4338-1

**Published:** 2016-10-11

**Authors:** Savinda Liyanagedara, Robert McLeod, Hassan A. Elhassan

**Affiliations:** 1 Cardiff University School of Medicine, Cardiff, UK; 2 ENT Department, University Hospital Wales, Cardiff, UK

**Keywords:** Exercise induced laryngeal obstruction (EILO), Continuous laryngoscopy exercise test (CLE), CLE-test sums score, Laryngeal control therapy (LCT), Endoscopy supraglottoplasty (ES)

## Abstract

Exercise induced laryngeal obstruction (EILO) is a condition where inappropriate vocal cord or glottic closure occurs during exercise. This review of the literature provides an overview of the current understanding of the definition, epidemiology, diagnosis and management of EILO. Using The Preferred Reporting Items for Systematic Review and Meta-Analyses (PRISMA) guidelines the Cochrane, Embase, Ovid MEDLINE and PubMed databases were searched. Four search domains “exercise”, “induced”, “laryngeal” and “obstruction” were used. Primary searching found 469 records, 308 were excluded following screening of titles and citation. 100 were duplicates, a further 47 studies were excluded after applying inclusion and exclusion criteria. Two studies were identified following cross-referencing. A total of 15 studies were included. The last search date was 6/06/15. Average prevalence in the general adolescent population and athletes was 7.1 and 35.2 %, respectively. Dyspnoea was reported in 96.5, 99 and 100 % of three EILO patient cohorts. Two studies (*n* = 107) reported continuous laryngoscopy during exercise (CLE) testing could differentiate between patients and controls. In two studies (*n* = 33) the visual analogue scale (VAS) showed a beneficial effect of endoscopic supraglottoplasty (ES). Thirty-eight out of 43 patients who received two or more laryngeal control therapy sessions (LCT) had improvement or resolution of EILO symptoms. Exercise induced dyspnoea is the most common EILO symptom. EILO has a high occurrence in adolescents and athletes. The CLE test is the current gold standard for EILO diagnostics. Management of EILO includes both surgical and non-surgical interventions.

## Introduction

Laryngeal lumen size is dependent on its cartilaginous skeleton, neuromuscular control of the vocal cords and aryepiglottic folds [1]. Physiologically during exercise the larynx adjusts via abduction of the vocal folds and aryepiglottic folds. Occasionally, during exertion laryngeal obstruction can occur at the supraglottic or glottis level [2, 3]. Reasons for this could be one or a combination of laryngeal pathophysiology, aerodynamic mechanisms and psychodynamic causes [1]. The varied underlying causes has led to numerous terms, namely exercise induced vocal cord dysfunction (EI-VCD), exercise-induced laryngomalacia (EIL), exercise induced vocal cord dysfunction (EIVCD) and exercise induced paradoxical vocal fold motion (EIPVFM), being used to describe the condition [4–6]. EI-VCD, EIL, EIVCD and EIPVFM are collectively referred to—by the umbrella term—exercise induced laryngeal obstruction (EILO) [1]. In this paper specific conditions under the umbrella of EILO will be mentioned when necessary.

EILO is believed to be prevalent amongst the adolescent population particularly athletes [3, 7]. No consensus regarding other demographics and relative prevalence between the sexes exists [7]. EILO is characterised by exercise-induced stridor, a harsh inspiratory sound due to turbulent airflow through a narrow laryngeal opening. EILO stridor symptoms typically peak towards the close of an exercise session and throughout the first 2–3 min of recovery [1]. Other symptoms include respiratory distress, prolonged inspiration, hyperventilation attacks, and frank panic reactions.

Continuous laryngoscopy during exercise test (CLE test) is currently the gold standard diagnostic test for EILO [3, 6, 8–10]. During CLE the patient runs to symptom limiting distress or to exhaustion on a treadmill wearing a facemask connected up to a cardiopulmonary exercise unit and a 12 lead ECG. Simultaneous flexible nasal laryngoscopy is performed and a microphone records respiratory sounds. Gas exchange: minute ventilation (number of breaths in a minute), tidal volume (volume of air breathed in and out during normal breathing), peak VO2 (the maximum rate of oxygen consumption as measured during incremental exercise), respiratory quotient (the ratio of the volume of carbon dioxide evolved to that of oxygen consumed in a given time) and tidal exercise flow volume loops are also measured. The test is positive if the patient reproduces their laryngeal symptoms, ideally supported by a plateau in oxygen consumption and/or the heart rate response [1, 2].

Provocation in CLE testing is any form of physical exercise that can potentially reproduce the clinical symptoms of EILO. Exercise provocation with maximal physical stress test (MPS) is widely used in CLE but it has been criticised for the lack of standardisation and reproducibility. For this reason, novel approaches such as eucapnic voluntary hyperventilation (EVH), where subjects inhale atmospheric air with 5 % of CO_2_ to induce laryngeal obstruction have been investigated [4].

Objective analysis of the laryngoscopic images obtained from CLE, include the CLE-test scoring system and the EILOMEA software [11, 12]. The CLE-test scoring system assesses the degree of medial rotation of the aryepiglottic folds and adduction of the vocal folds scored at two distinctive periods in time during the exercise period, i.e. at moderate exertion (when beginning to run on the treadmill) and at greatest effort (shortly before exhaustion). Adduction is scored from 0 (neutral position or abduction) to a highest grade of 3. Such a method creates four sub-scores ranging from 0 to 3, i.e. glottic and supraglottic adduction at moderate—(A and B), and maximal—exercise (C and D). The sum score (E) is then classified into three groups: 0–2 (I), 3–4 (II) and ≥5 (III) [1, 11]. EILOMEA involves the operator marking out laryngeal structures, i.e. the position of the vocal cords from a CLE still frame. The program then calculates a series of cross-sectional areas and distances to diagnose EILO [12].

Both non-surgical and surgical interventions exist for EILO. As the aetiology of EILO is heterogeneous, the management is personalised by taking into account the functional and anatomical findings in each case.

Psychotherapy, speech therapy, and injection with botulin toxin and inhaled ipratropium have been used as non-surgical treatment options [1]. Laryngeal control therapy (LCT) is a psychologically mediated therapy and focuses on breathing with lower abdominal movement, audible nasal sniffing on inhalation, and controlled exhalation through the mouth. LCT aims to increase awareness of how to make the glottic aperture bigger, perform slow breathing and increase the force on exhalation [8].

The surgical treatment for EILO called endoscopic supraglottoplasty (ES) involves incising the aryepiglottic folds anterior to the cuneiform cartilages and partial removal of mucosa from the top of the tubercles [13].

This review aims to identify and evaluate the clinical presentation, diagnosis and treatment of EILO.

## Systematic method and results

### Search strategy

Using the Preferred Reporting Items for Systematic Review and Meta-Analyses (PRISMA) guidelines, the Cochrane Database, Embase (1947–present), Ovid MEDLINE (1946-August Week 2 2014) and PubMed (1966–present) databases were used to carry this systematic review. Four search domains were used and combined using “AND”, and terms within each domain was combined by “OR”. The first domain included “exercise”, “rower”, “exertion”, “functional”, “exertional”, “rowing”, “high-level exercise”, “exercise-induced”, “exercising”, “athletes”, “elite athletes”, “paradoxical”, “episodic”, “paroxysmal”, “maximal exercise”, “treadmill”, “high speed treadmill exercise” and “high-intensity exercise”. The second domain used the terms “Induced”, “inducible”, “stress-inducible” and “stimulating provoked”. The third domain included the terms “laryngeal”, “vocal cord”, “vocal-cord”, “larynx”, “glottis configuration”, “vocal fold”, “upper respiratory tract”, “laryngomalacia”, “soft palate”, “voice”, “upper airways”, “glottis” and “laryngospasm”. The final domain included the terms “obstruction”, “dysfunction”, “dyspnoea”, “bottleneck”, “stridor”, “obstructions”, “dyspnea”, “upper airway function”, “asphyxia”, “aphonia”, “inspiratory”, “obstruction”, “vocal cord adduction”, “laryngochalasia”, “vocal cord motion”, “motion”, “laryngeal dyskinesis”, “extrathoracic upper airways obstruction”, “airflow mechanics”, “vocal fold movement” and “laryngeal paralysis”. The last search date was 6/06/15.

**Fig. 1:**
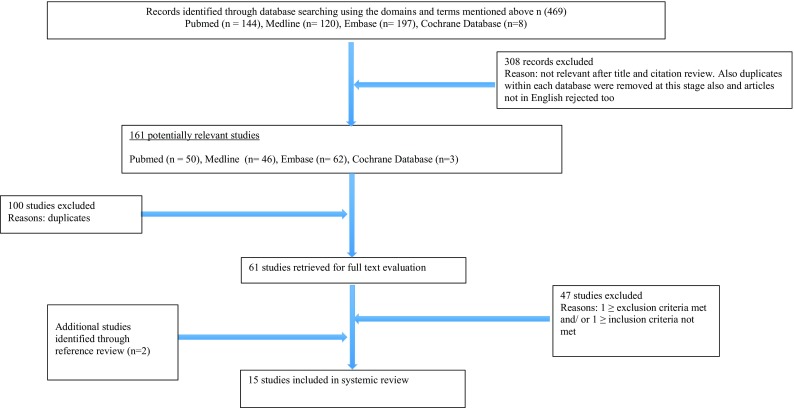
Search strategy to obtain literature is presented. Articles on EILO were included. Exclusion criteria include reviews, thesis, case reports, opinion-based reports, letters, web pages, congress abstracts, Small studies (<5 participants), animal studies and cadaver studies

The primary search found 469 records (Fig. 1). Three hundred and eight of these were excluded following screening of the title and abstract for relevant articles related to exercise induced laryngeal obstruction. One hundred of the remaining studies were duplicates, leaving 61 studies for full text evaluation. After applying inclusion and exclusion criteria to these full texts, a further 47 studies were excluded. Two further studies were identified following cross-referencing. A total of 15 studies were included.

## Review

### Clinical presentation

Dyspnoea is the most common symptom of EILO, with percentages of dyspnoea being 99, 100 and 96.5 % in the cohorts with 270 patients [6, 8, 9]. Hoarse voice and/or stridor were common symptoms found in 54 % of EILO patients [1, 6, 8, 9].

EILO is thought to be prevalent amongst adolescents and particularly athletes [3, 7]. However, these studies (Table 1) did not compare prevalence of EILO in athletes to either the general population or a control population. For this reason, it is not possible to say with certainty if EILO principally affects adolescents and athletes; these populations maybe physically more active hence more likely to suffer from EILO. Additionally, our search did not yield any studies on the prevalence of EILO in the general population. Studies looking into prevalence within the general public are required as they serve as a point of comparison for studies on athletes and adolescents, thus could confirm if this is indeed a condition particularly affecting those cohorts.

**Table 1:** Prevalence of EILO

Study	No. of subjects	Age group of cohort	Prevalence of EILO/EIVCD/EIPVFM (%)
Christensen et al. [11]	556	14–24	42 (7.5)
Nielsen et al. [3]	88	Median age of 17	31 (35.2)
Johansson et al. [5]	146	12–13	8 (5.5)
Total	790	–	81 (10.25)

*EILO* exercise induced laryngeal obstruction, *EIVCD* exercise induced vocal cord dysfunction, *EIPVFM* exercise induced paradoxical vocal fold motion

The average prevalence in the general adolescent population is 7.1 % (*n* = 706) [5, 7]. Eighty-eight adolescent athletes with a mean age of 17 reported a prevalence of 35.2 %. This high prevalence of EILO (35.2 %) may be due to selection bias, as athletes were referred to a tertiary referral clinic specialising in asthma and sports [3]. The low participation rates in one study may result in underestimation of the EILO prevalence (17.6 %) [5].

Data extracted on prevalence of EILO is in Table 1.

It is known that the size of the larynx is almost the same in boys and girls till puberty and after puberty the anterior-posterior diameter of the larynx virtually doubles in males [10, 11]. A larynx with a wider diameter would mean that there would be a lower chance of obstruction. As such it is hypothesized that EILO will have equal prevalence between males and females at a pre-pubertal age and a higher prevalence at a post-pubertal age and the data for the prevalence of EILO in males and females supports this. Two studies found a higher prevalence of EILO in females. One of these studies identified a female/male odds ratio of 3.41 in EILO and this was in an older population (14–24) allowing most of the male subjects to have an adult size larynx (by the age of 16–17) [7]. The second study reported a higher prevalence of EILO in female athletes (OR = 4.09) at a primarily post-pubertal age [3]. A final study reported equal prevalence of EILO regardless of sex in participants of a pre-pubertal age [5].

### Differential diagnoses

The symptoms of EILO and exercise induced bronchoconstriction share many similarities and often the two conditions can co-exist [8]. The rates of coexistent asthma have been as high as 56 %, as such clinicians should have a high clinical suspicion of EILO when exercise induced asthma symptoms are present [3, 14] (Table 2).

**Table 2:** Symptoms/features of EILO

Study	Number	% Dyspnoea during exercise	% Hoarse voice/stridor during exercise	% Cough during exercise	% Dysphagia during exercise	% Open glottic configuration during exercise
Chiang et al. [8]	104	99	43	38	22	–
Olin et al. [9]	23	100	56.5	–	–	78.3
Tilles et al. [6]	143	96.5	50	9.8	–	–
Røksund et al. [1]	113	–	68	–	–	–
Overall	383	98	54	22	22	78.3

*EILO* exercise induced laryngeal obstruction

### CLE testing and scoring

CLE test was successful in inducing or correlating with symptoms of EILO (*n* = 258) [1, 10, 11]. A CLE test on 27 subjects with a history of dyspnoea on exercise found nine to have EILO. Additionally, this test was well tolerated and can be easily performed [10]. It has been reported that a positive correlation between CLE-test sum score and symptom score (*P* < 0.001) exists, meaning that the CLE-test sum score could be reliably used to evaluate laryngeal function during exercise [11]. Finally, a typical laryngeal response pattern to exercise can be visualized in a large quantity of patients with suspected upper airway obstruction. This is because severe adduction of laryngeal structures correlated with inspiratory distress in 113 (75 %) symptomatic patients [1].

A CLE test performed on 15 controls found normal laryngeal function with no subjective symptoms of dyspnoea [10]. Furthermore, significant differences of CLE-test sum score between patients and volunteers (*P* < 0.001) [11]. The amount of inter- and intra-observer agreement for each sub-score of the CLE test varied between 70 and 100 %, suggesting repeatability of the test [11]. Such findings support the ability of the CLE test to differentiate between patients and controls (*n* = 107) [10, 11].

Despite the literature suggesting the usefulness of CLE in diagnosing EILO, one should be aware that performance through the test, together with subjective perception of distress is likely to be affected by features such as skills, attitude, expectations, and personal goals. Regarding this point, it has been observed that young athletes who continued running regardless of harsh symptoms of distress, whilst different subjects stopped sooner with comparatively minor symptoms [11]. This is exemplified as certain patients had a low CLE-test score although a high CLE-test score would be anticipated due to their somewhat high score of subjective complaints. More studies are needed to clarify complex interactions between somatic and psychological factors, physical capacity, and motivation and how they may influence the test score [11].

### Provocation for CLE

93 subjects showed provocation was important in CLE testing, as sensitivity of the test without provocation was 52 % and with provocation it was 89 % [8]. Stationary bicycle and treadmill as methods of provocation have helped form CLE-tests with good diagnostic utility [1, 10]. However, the reproducibility of the test using the treadmill wasn’t assessed. Additionally, in the CLE test using the treadmill, patients’ subjective feeling of dyspnoea at the laryngeal level was not always accurate: six (40 %) of the patients, who reported subjective laryngeal dyspnoea, had a wide-open larynx when visualized via fiberoptic video-laryngoscopy [10].

EVH seems to be a better alternative to provocation than MPS in the CLE test. However, significantly fewer recordings were utilisable for assessment due to resilient mucus on the tip of the fiberoptic-laryngoscope in the EVH test compared to the MPS. These results were from a non-patient population of highly active athletes, so extreme cases of obstruction namely severe cases of arytenoid rotation (AR) were not observed. It, therefore, remains unknown whether the EVH test will induce severe AR in extreme cases. After addressing such issues in future studies and with an amended protocol allowing better visualisation of the larynx, we recommend having a CLE test with EVH as the method of provocation. This combination may allow for a more standardised and repeatable test, thus allowing more reliable comparison of results between subjects [7].

### EILOMEA

EILOMEA was shown to give the diagnosis and degree of obstruction objectively for EILO in 97 subjects. Additionally, EILOMEA can be used to distinguish an individual with no EILO from a person with mild or moderate EILO but can not be used to distinguish mild or moderate EILO from severe EILO [12]. The authors of this study mentioned that the reliability of EILOMEA could be made better. For example, the equipment utilised for recording was not of high quality. Additionally, the inter-rater reliability of the EILOMEA method could be improved [12].

Data on CLE testing, provocation for CLE and EILOMEA are in Table 3.

**Table 3:** CLE testing

Study	Number of patients	Number of controls	Diagnostic method	Aims	Methodology
Tervonen et al. [10]	30 (successfully carried out the test 27)	15	Fiberoptic videolaryngoscopy during bicycle ergometry	Develop and validate a new diagnostic method for EIVCD by combination of continuous fiberoptic laryngoscopy and bicycle ergometry test	Thirty consecutive patients referred to a laryngologist due to EIVCD suspicion and 15 healthy controls underwent CLE testing using fiberoptic videolaryngoscopy during bicycle ergometry
Maat et al. [11]	80	20	CLE	Develop and validate a scoring system for laryngeal obstruction as visualized during the CLE-test	80 patients and 20 symptom-negative volunteers performed a CLE test. Every participant scored symptom severity during exercise. The scoring system has four sub-scores, each graded from 0 to 3. Two independent laryngologists, blinded to clinical data, did scoring of the video recordings of the larynx twice. Assessment of the inter- and intra-observer agreement proportions for each sub-score through these four sessions was done
Christensen et al. [12]	97	–	EILOMEA	EILOMEA is diagnostic software used to objectively describe images gained by CLE test. Evaluation of this software was performed to evaluate the reproducibility and clinical applications of this tool for the diagnosis of EIL and EIVCD	Nighty seven subjects aged between 14 and 24 had CLE testing performed. An expert assessed the severity of EIL and/or EIVCD for each laryngoscopic recording and compared this with data from EILOMEA
Chiang et al. [8]	93	–	FFL	Single institution retrospective review and cohort analysis was performed to review the diagnostics and treatment of EPVFM	Single-institution retrospective review identifying patients with EPVFM was done. FFL performed on these patients were reviewed with regarding the presence of laryngeal pathology and the presence of PVFMD at rest and/or with exertion. Symptom outcomes were graded as complete resolution, improvement, or unchanged following therapy
Roksund et al. [1]	151	20	CLE	Aimed to study transnasal laryngoscopic evaluation of laryngeal function during treadmill exercise	

*CLE* continuous laryngoscopy during exercise, *FFL* flexible fiberoptic laryngoscopy, *EIL* exercise induced laryngomalacia, *EIVCD* exercise induced vocal cord dysfunction, *EPVFM* exercise induced paradoxical vocal fold movement, *PVFMD* paradoxical vocal fold motion disorder

### Flow volume loops

Flow volume loops in isolation are ineffective for diagnosing EILO (*n* = 123) [4, 9]. Eight of 23 patients with exercise-induced stridor had no evidence of inspiratory limitation by flow volume loop analysis. Eleven of 23 patients had open glottic configuration and audible stridor, of these five patients had no evidence of inspiratory limitation by flow volume loop analysis to suggest EILO. Additionally, low inter-rater agreement regarding flow volume loop analysis (52 % kappa 0.33) [9].

There is no significant connection between the laryngoscopic findings and the flow-volume data with no agreement between the four physicians in their assessment of the flow-volume loops (kappa <0.00) and no significant correlation between physician’s assessments and laryngoscopic findings [4].

Data extracted on investigations other than CLE is provided in Table 4.

**Table 4:** Other diagnostic methods compared to CLE

Study	Number of patients	Number of controls	Diagnostic methods	Aims	Methodology
Christensen and Rasmussen [4]	39	–	EVH compared to CLE	To assess if a EVH test can produce laryngeal obstructions laryngoscopically identical in subtypes and development as seen through an exercise test	EVH and CLE testing was done during the screening of two national athletic teams(* n* = 67). Laryngoscopic recordings were examined for usability, abnormalities and maximal supraglottic and glottic obstruction using Eilomea and CLE-score. The questions on ERRS were asked to each participant, and if symptoms that occured during each provocation matched those which occured during regular training. A total of 39 completed both tests
Christensen et al. [15]	100	–	FVL compared to CLE	To compare physician assessed pre- and post-exercise flow-volume loops and flow data with laryngoscopic findings during exercise	Data from 100 consecutive CLE-tests were analysed. Laryngoscopic images were compared with the corresponding pre- and post-exercise flow-volume loops assessed by four separate physicians
Olin et al. [9]	23	–	FVL	The objective of this study was to highlight a group of patients who demonstrated important clinical findings of EIPVFM (exertional dyspnea with audible stridor) without simultaneously definitive physiologic findings (mild glottic adduction and normal flow volume loops)	We reviewed the records of 23 patients who performed continuous laryngoscopy during exercise. 3 blinded physicians independently evaluated isolated audio tracks, video tracks, and flow volume loops of the patients for stridor, glottic configuration, and the presence of inspiratory limitation on exercise flow volume loops at peak work capacity

*EVH* eucapnic voluntary hyperventilation, *FVL* flow volume loops, *EIPVFM* exercise induced paradoxical vocal fold movement, *EILO* exercise induced laryngeal obstruction, *CLE* continuous laryngoscopy during exercise

### Non surgical treatment

Psychotherapy, speech therapy, and injection with botulin toxin and inhaled ipratropium have been used as non-surgical treatment options [1]. Speech therapy has been associated with more than 80 % success [16]. More studies are required to evaluate these approaches [1]. Laryngeal control therapy (LCT) is effective for treating EILO. Of the 43 patients who received two or more LCT sessions, 38 (88 %) had improvement or complete resolution of symptoms and 20 were discharged [8].

### Surgical treatment

The site of EILO is important as it guides treatment choice. The anatomical location of obstruction appears to occur principally in the supraglottic part of the larynx [1, 3]. In a cohort of athletes 71 % of the obstruction was at supraglottic level [3].

Two studies (*n* = 33) on ES mentioned its effectiveness. The visual analogue scale (VAS) results for both studies showed a beneficial effect of surgery [2, 13]. One of these studies showed subjective improvement of breathing difficulties [13]. In the other study symptoms improved in the surgically treated group from 87 to 25 on the VAS and on follow up. Surgically treated patients reported lower level of complaints, higher ability to perform exercise compared to conservatively treated patients (*P* < 0.001). The CLE scores normalized for 84 % of the surgically treated patients, this was significantly more than conservatively treated patients. Using objective measures of FEV1, VO/kg, respiratory quotient and VE max before and after surgery, we performed a paired *t* test. The paired *t* test showed that there was no significant change pre and post operatively on these values [2]. This finding may be due to a lack of power as the study only included ten subjects or short observation period (3 months). This study also showed that surgical treatment at the supraglottic level in EILO patients happens to have a positive effect on obstruction at the glottic level as well, but the reasons for this are not known [2]. Although there is not enough evidence for a surgical intervention for individuals with glottic obstruction, for patients with supraglottic obstruction endoscopic supraglottoplasty shows a statistically significant improvement in EILO symptoms and can be recommended in adult patients who have failed conservative or non-surgical management.

In paediatric patients, it is hypothesised that increased laryngeal diameter due to growth might spontaneously improve their exercise capacity [2]. For this reason, a conservative approach may be recommended, though these patients as a whole report persisting symptoms at follow-up. That laryngeal function had normalized in only three out of 14 re-tested control patients argues against such a hypothesis [2]. Two paediatric studies showed an improvement with surgery and did not document any adverse effects of surgery. As there is little evidence for the hypothesis of natural resolution of EILO with time clinicians should consider surgery as a treatment modality, where conservative non-surgical methods have failed [2, 13]. Data on investigations/treatments is provided in Table 5.

**Table 5:** Treatment of EILO

Study	Number of patients	Number of controls	Treatment method	Aims	Methodology
Maat et al. [13]	10	–	ES with laser incision in both aryepiglottic folds anterior to the cuneiform cartilages and removal of the mucosa around the upper parts of the tubercles	Evaluate the usefulness of the CLE-test as a method for selecting patients for surgical intervention and evaluating treatment effects postoperatively	Ten patients underwent ES. CLE- test was done on each patient before and 3 months after surgery
Chiang et al. [8]	96	–	LCT	The aim was to see of LCT was an effective treatment for EPVFMD	Patients diagnosed with PVFMD via FFL as well as symptoms with exercise were selected. Therapy was reviewed and symptoms outcomes were graded as complete resolution, improvement or unchanged following therapy
Maat et al. [2]	23 surgically treated patients with (ST)	71 conservatively treated patients with breathing exercise (CT)	Laser supraglottoplasty	Reveal the natural history of supraglottic EILO and compare the symptoms, and the function of the larynx in conservatively versus surgically treated patients	Follow-up study of supraglottic EILO was performed. In 94 patients with predominantly supraglottic obstruction a questionnaire-based survey was conducted 2–5 years after EILO diagnosis via CLE test. Seventy-one patients had CT and 23 had ST. A second CLE test was carried out in 14 CT and 19 ST patients

*ES* endoscopic supraglottoplasty, *LCT* laryngeal control therapy, *ST* standard therapy, *CT* control therapy, *EILO* exercise induced laryngeal obstruction, *FFL* flexible fiberoptic laryngoscopy, *EPVFMD* exercise induced paradoxical vocal fold motion disorder, *CLE-test* continuous laryngoscopy during exercise test

**Fig. 2:**
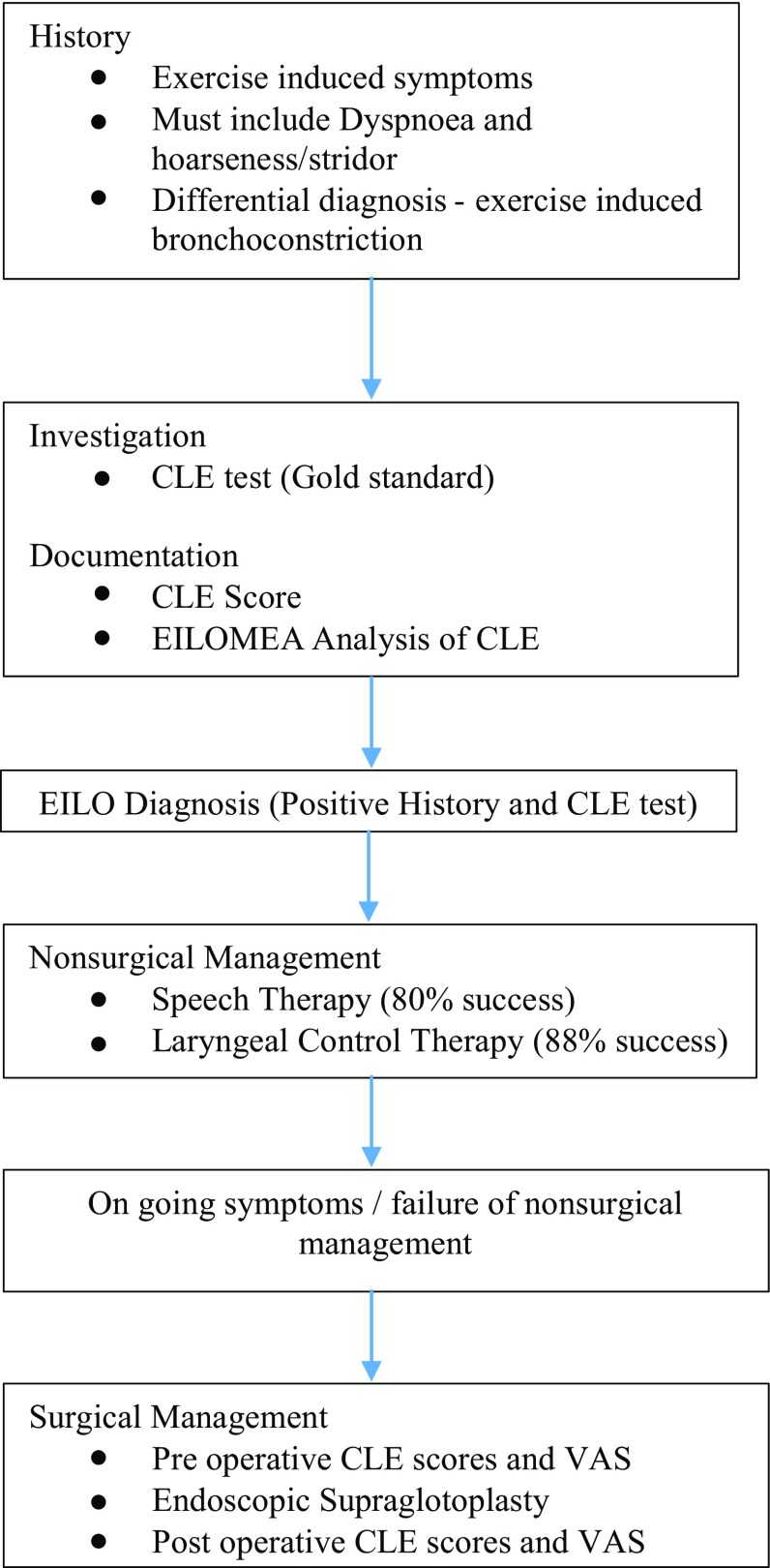
Algorithm for the diagnosis and treatment of EILO

We have devised an algorithm for the diagnosis and treatment of EILO based on the findings of this review (Fig. 2).

## Conclusion

This systematic review looked at the available literature on EILO symptoms, prevalence, demographics, diagnostics and treatment. Common symptoms of EILO must include exercise induced dyspnea and stridor or hoarseness. Minimal prevalence data on EILO in the general population exists; classically it was thought to affect adolescents and athletes but these studies contained selection bias. The gold standard for diagnosis is the CLE test with both the CLE-test sum score and EILOMEA as valid ways of objectively analysing the test. Speech therapy Laryngeal control therapy was highly effective with 89 % of patients having symptom resolution or improvement. Endoscopic laser supraglottoplasty has been shown to be effective in selected cases and can be recommended where non-surgical management has failed.
